# Neural Evidence for Action-Related Somatosensory Predictions

**DOI:** 10.1523/JNEUROSCI.0889-25.2026

**Published:** 2026-07-23

**Authors:** Caoimhe Moran, Hinze Hogendoorn, Ayelet N. Landau

**Affiliations:** ^1^Melbourne School of Psychological Sciences, The University of Melbourne, Parkville, Victoria 3010, Australia; ^2^School of Psychology and Counselling, Queensland University of Technology, Brisbane, Queensland 4000, Australia; ^3^The Department of Psychology and the Department of Cognitive and Brain Sciences, The Hebrew University of Jerusalem, Jerusalem 91905, Israel; ^4^Department of Experimental Psychology, University College London, London WC1E 6BT, United Kingdom

**Keywords:** action, EEG, MVPA, prediction, tactile

## Abstract

The tactile consequences of self-initiated movements are thought to be predicted by a forward model (FM), yet the precise neural implementation of these predictions remains unclear. In nonmotor contexts, expectations are thought to activate sensory neurons tuned toward the expected stimulus. This acts as a predictive template against which afferent sensory input is compared. It is unclear whether FM predictions have a similar neural instantiation. Here we employed time-resolved multivariate decoding on human electroencephalography during self-generated movements to examine the content of predictive neural activity. Human participants (males and females) performed index finger movements which were predictably paired with a vibration to either the index or ring finger of the opposite, passive hand. On some trials the tactile stimulus was unexpectedly omitted. Results revealed above-chance finger decoding in the premovement period supporting a predictive representation of expected stimulation location. As the movement approached, this predictive activity became similar to late-stage processing of a physical tactile stimulus. On omission trials, we found that despite the absence of afferent input, finger location could be decoded ∼120 ms after expected stimulus onset. This shows a stimulus-specific omission response. Together these findings indicate that self-generated movement preactivates neurons tuned toward expected tactile consequences.

## Significance Statement

Engaging effectively with the world relies on our ability to anticipate the sensory consequences of our own movements. To characterize how the brain encodes action-driven tactile predictions, we recorded electroencephalography (EEG) while participants performed finger movements paired with vibrations to either the index or ring finger of the opposite hand. Crucially, we introduced unexpected omissions, where no stimulus followed the movement. Applying time-resolved EEG–decoding methods, we show that premovement neural activity encodes expected stimulation location. In addition, on omission trials, despite the absence of bottom–up stimulation, we could decode expected stimulation location. Such predictions of self-produced tactile events likely ensure accurate dissociation of self from other and promote a sense of agency over our own motor actions.

## Introduction

Every movement comes with an expectation of its sensory consequences. When you kick a ball, you anticipate the feeling of impact against your foot and the sight of the ball moving through the air. These expectations act as templates against which incoming sensory information is compared (Summerfield and Egner, 2009; de Lange et al., 2018), termed action-effect predictions (James, 1981).

Major theories propose that the brain uses a forward model (FM) to predict the consequences of self-produced actions (Blakemore et al., 1999; Dogge et al., 2019; Kilteni and Ehrsson, 2020, 2022). As a motor command is issued, a copy of this command, known as corollary discharge, is sent to the FM; which simulates body movements to anticipate the sensory effects (Todorov, 2004). Consistent with this framework, previous work has shown that voluntary actions modulate activity in the sensory cortex prior to expected outcomes, as well as earlier motor-related signals such as the readiness potential (Reznik et al., 2018, 2021; Gärtner et al., 2025). Sensory input is compared with predictions, enabling a Bayes-optimal estimate of action-effects. Errors made when predicting afferent inputs are exploited to update internal models, refining future predictions. By their nature, self-generated actions have highly predictable consequences resulting in minimal prediction error (PE). This mechanism explains attenuation of self-generated action consequences in vision (Hughes and Waszak, 2011; Straube et al., 2017), audition (Baess et al., 2009; Weiss et al., 2011; Nelson et al., 2013; Schneider et al., 2018), and touch (Blakemore et al., 1999, 2000; Shergill et al., 2003; Bays et al., 2005, 2006; Hesse et al., 2010; Shergill et al., 2013; Kilteni et al., 2019; Kilteni and Ehrsson, 2020, 2022; Job and Kilteni, 2023).

In contrast, prediction violations enhance neural activity. The mismatch negativity (MMN), an ERP component indexing prediction violations (Alho, 1995), is regarded as the neural correlate of PE (Waszak and Herwig, 2007; Korka et al., 2019). Attenuation and the MMN are used as evidence of predictive coding (Clark, 2013), however, alternative explanations, including neural adaptation, cannot be ruled out (May and Tiitinen, 2001; Jääskeläinen et al., 2004; May and Tiitinen, 2010). Moreover, neural modulation by expectation provides only indirect evidence for predictive processes (Schröger et al., 2015). A more direct measure requires removing afferent input entirely to isolate prediction-driven neural responses from bottom–up activity (Arnal and Giraud, 2012).

Stimulus-omission paradigms may offer a more direct insight. When an action consequence is unexpectedly omitted, sensory-like ERP responses are produced (SanMiguel et al., 2013a; Dercksen et al., 2020, 2022; Korka et al., 2020). These responses occur only when stimulus omissions are rare, suggesting they reflect the PE (SanMiguel et al., 2013a). Omission paradigms remain underexplored in somatosensation. However, a recent study reported an electroencephalography (EEG) omission response to violated somatosensory action-effect predictions (Dercksen et al., 2024). Yet, traditional ERPs do not reveal whether the error response contains specific information (e.g., location, intensity, color) about an expected stimulus or whether it simply signals that an anticipated event failed to occur.

An essential aspect of tactile prediction is spatial specificity. Predicting where a touch will occur is critical for distinguishing self-generated from externally generated sensations and for establishing a sense of agency (Haggard and Tsakiris, 2009). While auditory studies have examined the specificity of prediction-related neural activity (SanMiguel et al., 2013a; Korka et al., 2020), to our knowledge, identity-specific somatosensory predictions during action remain largely unexplored.

In the present study, we used time-resolved EEG decoding to examine the spatial specificity of movement-driven tactile predictions. Participants performed self-timed finger movements followed by predictable tactile stimulation to the index or ring finger of the passive hand, with occasional omissions. We hypothesized that movement preparation triggers a finger-specific tactile prediction in the somatosensory cortex. Omitting the expected stimulus should generate a PE from the mismatch between prediction and absent input. Thus, above-chance decoding on omission trials would reflect predictive activity. We also expect above-chance prestimulus finger decoding, consistent with anticipatory sensory templates.

## Materials and Methods

### Participants

Data were collected from a total of 39 participants, of which 4 were excluded due to poor EEG classification performance (<52% average decoding accuracy when classifying finger location on passive trials). This exclusion criterion ensured that a reliable neural finger location signal could be decoded from each participant's EEG data, enabling meaningful interpretation of the cross-decoding analyses. Importantly, this procedure is not circular, as the criterion was applied exclusively to passive trials, while the reported conclusions are based on active trials. After exclusion, 35 participants remained (23 females; age range, 19–35; mean age, 24; SD, 3 years; 5 left-handed). All observers provided informed consent before beginning the experiment and were compensated with credit points or reimbursed 13 USD per hour. The project was approved by the local ethical committee.

### Apparatus and stimuli

The experiment was conducted in MATLAB Version R2021b on a BenQ XL2420Z monitor. The experiment was programmed using the Psychophysics Toolbox extension (Brainard, 1997; Pelli, 1997; Kleiner et al., 2007). A black fixation cross was presented in the middle of the screen at ∼60 cm from participants' eyes. Stimuli were delivered using TDK PowerHap Piezo Actuators (9 × 9 × 1.1 mm) connected to a piezo driver (Boreas Technologies, BOS1901 Development Kit). The piezo driver appears as a normal audio output to the computer. Thus, tactile stimuli were created by programming an audio sine wave which produced a vibration for 40 ms at 300 Hz. An infrared motion tracker (Leap Motion Controller using the Matleap MATLAB interface) was used to track participants' finger movements. When the distal phalange (the tip) of the index finger descended 20 mm from the starting position at trial onset, the target stimulus was delivered. Pilot testing ensured that the stimulus felt like an immediate consequence of the movement. Participants' hands were visually occluded during the experiment, and white noise was continually played through headphones to obscure the sound of the tactile stimulus. A test was run prior to experiment onset to ensure participants could not hear the vibration. The white noise volume was adjusted until the stimulus sound was fully obscured.

### Experimental design

**Figure 1.**
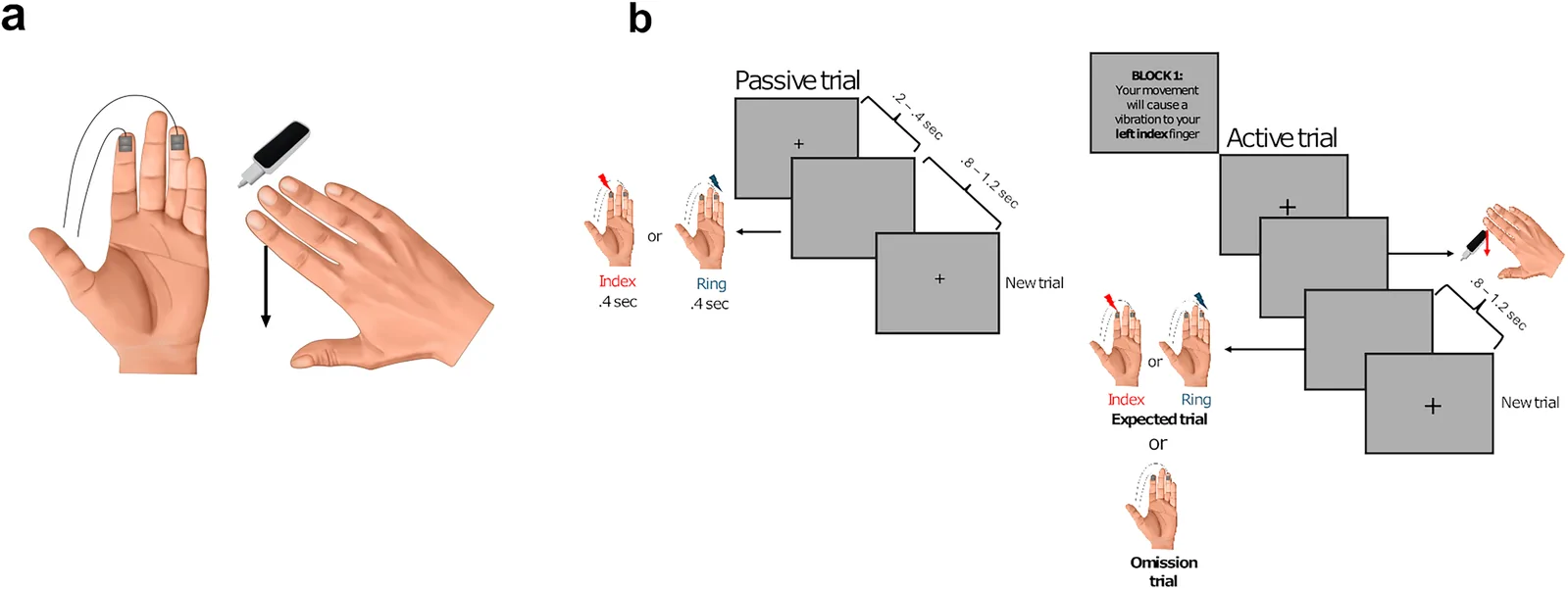
Paradigm and trial types. ***a***, Position of the hands during the experiment. Two tactors were attached to the left index and ring finger. Participants positioned their right hand above the motion sensor camera. The right hand remained still on passive trials, while in active trials, participants made a downward movement with the index finger. ***b***, Trial details for passive trials (left side) in which participants' both hands remained still. They received short vibrations (40 ms) to either their left index or ring finger with a jittered intertrial interval. Active trials (right side) required participants to move their right index finger downward. Depending on the block, they received a vibration to their left index or ring finger (expected trials) immediately after the movement. On 20% of trials, the vibration was omitted entirely (omission trials).

Participants were seated in a dimly lit room, their left hand, palm up, on the table (Fig. 1*a*). Tactors were placed on the apex of the left index and ring fingers. Their right arm lay on an armrest, palm face down, with the hand extended over the edge. The motion tracker was placed directly under the right hand (15 cm from the palm). After setup, participants received verbal and written task instructions. Depending on the block type (passive or active), participants were told to keep their right hand still or move their index finger downward. Under both conditions, participants were told to press a foot pedal with their right foot when they detected a tactile vibration at a lower intensity than the others. In addition, the experimenter demonstrated the correct hand positions and showed the participant how to accurately perform the movement. To ensure accurate calibration between the motion camera and movement, participants performed 10 index finger movements and received feedback on whether the movement was recorded or not. Subsequently, participants did a short practice in which their movement was followed by the tactile stimulus.

Participants completed passive and active blocks. On passive blocks participants looked at the fixation cross at the center of the screen and remained still. A tactile vibration was delivered to either the left index or ring finger for 40 ms. A jittered interval between 800 and 1,200 ms separated each trial and the fixation cross was removed from the screen with each stimulation and presented again at the beginning of the next trial (Fig. 1*b*).

In active blocks, cued by the appearance of a fixation cross, participants made a downward movement with their right index finger. The fixation cross was removed from the screen once initial movement was detected. This was immediately followed by a brief vibration (40 ms) to either the left index or ring finger, termed Active-Standard (AS) trials or omitted entirely, termed Active-Omission (AO) trials. Similar to passive trials, a jittered interval of 800–1,200 ms was introduced between stimulus delivery and the reappearance of the fixation cross, which signaled the start of the next trial. Within each active block, a fixed mapping between movement and vibration location was maintained, and the specific action-effect pairing was randomly assigned at the start of each block (i.e., movement paired with either index- or ring-finger stimulation).The stimulus was never delivered to the alternate finger within a block (Fig. 1*b*).

In total, there were five passive blocks with 120 trials in each block and six active blocks with 80 trials in each block. Omission trials constituted 20% of active trials. Across both active and passive blocks, participants were asked to detect and respond to tactile stimuli at a lower intensity by pressing a foot pedal. These made up 7.5% of trials (6 trials per active block and 10 trials per passive block) and were removed from the main analysis. This task ensured that participants were paying attention to the tactile stimulus but not specifically that it was delivered to the index or the ring finger, which are the conditions that we use for the decoding analysis. All blocks of the same type (passive or active) occurred consecutively. The block order was counterbalanced across participants such that participants completed either the active or passive blocks first.

### EEG acquisition and preprocessing

EEG was recorded throughout the experiment as participants performed the passive and active trials. A g.GAMMAcap (gTec) and a g.HIamp amplifier (gTec) were used. The 62 active electrodes on the cap were positioned according to the extended 10–20 system. Two active electrodes attached to the earlobes served as the reference. In addition, we recorded the horizontal and vertical electrooculogram using passive electrodes placed at the outer canthi of either eye and above and below the left eye, respectively. EEG was continuously sampled at 512 Hz.

All preprocessing steps were performed using the FieldTrip toolbox (Oostenveld et al., 2011) in the MATLAB environment. First, the EEG data were referenced offline to the average of the earlobe electrodes. Then, data were inspected visually [using the function ft_databrowser()], and bad channels of each participant were rejected. Rejected channels were replaced with a spherical spline interpolation [using the “spline” method in ft_channelrepair()]. Scalp muscle artifacts were detected as epochs in which the amplitude of the bandpass-filtered signal at 110–140 Hz at all channels exceeded a *z* score of 20, with the function ft_artifact_zvalue(). All preprocessing was done on the session as a whole (i.e., without dividing into trials). Division into trials was done for each of the subsequent analyses separately and remaining noisy trials were removed.

Across all trial types, epochs were time-locked to the onset of the tactile stimulus, separately for passive, AS, and AO trials. To examine normal stimulus processing, epochs were extracted from 200 ms before to 800 ms after stimulus onset for passive and AS trials. To test for omission trial effects, the same epoch was extracted around an expected stimulus onset (which was not presented) using AO trials. In another analysis, to look for possible prestimulus effects, AS trials were epoched from 300 ms before stimulus onset to 800 ms after. Across all analyses, trial segments were baseline corrected to the mean of the 100 ms period before actual or expected stimulus onset.

### Multivariate pattern analysis

All multivariate pattern analyses (MVPAs) were performed in Python (V 3.11.3). We used a selection of electrodes over the somatosensory and motor cortex (“FT7,” “FC5,” “FC3,” “FC1,” “FCz,” “FC2,” “FC4,” “FC6,” “FT8,” “T7,” “C5,” “C3,” “C1,” “Cz,” “C2,” “C4,” “C6,” “T8,” “TP7,” “CP5,” “CP3,” “CP1,” “CPz,” “CP2,” “CP4,” “CP6,” “TP8”) to train linear discriminant analysis (LDA) classifiers (Grootswagers et al., 2017). This decision was motivated by the fact that the noise artifact from tactile stimulation was strongest in the frontal electrodes and the occipital electrodes are not relevant for the research questions. Each classification analysis was performed at the level of single subjects. It is possible that a similar pattern is elicited by the expectation of touch and actual physical stimulation, but this may occur at different times. Thus, we used temporal generalization methods whereby a classifier is trained at each timepoint of the training set and tested on each timepoint of the test set. This provides a decoding matrix where each cell represents the average classification accuracy at that train-test timepoint. If the representation of finger location evolves similarly in both train and test sets, above-chance decoding will occur along the diagonal. On the other hand, if the representation unfolds differently over time across train and test sets, above-chance decoding will be off the diagonal.

### Within-condition decoding

First, we examined whether finger location could be decoded when training and testing within a condition (passive and AS). This involved training an LDA classifier on a portion of within-condition data and testing it on left-out independent trials across all timepoints, using a fivefold cross-validation method. This was done time-locked to stimulus onset (−200–800 ms). An above-chance classification performance indicates that the EEG signal contained information that allowed the classifier to discriminate between stimulation to the index or ring finger.

### Omission trial decoding

To test for finger-specific activity despite the absence of a stimulus, we ran cross-condition decoding analyses. Here, LDA classifiers were trained on passive or AS trials (−200 to 800 ms around stimulus onset) and tested on AO trials (−200–800 ms around expected stimulus onset). Cross-validation was not necessary because training and test sets are independent. We also ran an exploratory analysis to probe the effect of condition order on AO trial decoding. We split participants into two groups. The “Passive First” group included participants who completed the passive condition before the active condition and the “Passive Last” group included participants who completed the passive condition after the active condition. We then ran the MVPA on each group separately, training on passive trials and testing on AO trials.

### Prestimulus decoding

To test for the presence of stimulus-specific predictive activity, we trained our classifiers on a subset of AS trials including the prestimulus and poststimulus period (−300 to 800 ms) and tested them on the prestimulus period of left-out AS trials (−300 to 0 ms). Above-chance decoding along the diagonal in the prestimulus period indicates that the neural activity before stimulus onset contains information about finger location. Off-diagonal decoding, at poststimulus training timepoints, indicates that prestimulus activity is similar to stimulus-evoked activity.

### Statistical inference

We used Bayes factors (BFs) to determine above-chance decoding and at-chance decoding (i.e., null hypothesis) at every timepoint within each of the participants using the BF R package (Morey and Rouder, 2018) implemented in Python (Teichmann et al., 2022). We set the prior for the null hypothesis at 0.5 (chance decoding) for assessing the decoding results. A half-Cauchy prior was used for the alternative hypothesis with a medium width of *r* = 0.707. Based on Teichmann et al. (2022), we set the standardized effect sizes expected to occur under the alternative hypothesis in a range between −∞ and ∞ to capture above- and below-chance decoding with a medium effect size (Morey and Rouder, 2018).

BFs larger than 1 indicate that there is more evidence for the alternative hypothesis than the null hypothesis (Dienes, 2011) with a BF greater than 3 considered as “substantial” evidence for the alternative hypothesis i.e. decoding above- or below-chance level. A BF less than one-third is considered substantial evidence for the null hypothesis, i.e., chance-level decoding (Jeffreys, 1961).

## Results

### Task performance

Participants performed above chance in detecting the tactile stimuli presented at a lower frequency with a mean accuracy of 62% (SE, 14%) in active trials and 70% in passive trials (SE, 15%). Reaction times (RTs) were calculated from the time that the low-intensity stimulus was delivered until the time that the foot pedal was pressed. The mean RT was 714 ms (SE, 132 ms) in the passive block and 754 ms in the active block (SE, 43 ms).

### Within-condition decoding of stimulation location (index vs ring finger)

**Figure 2.**
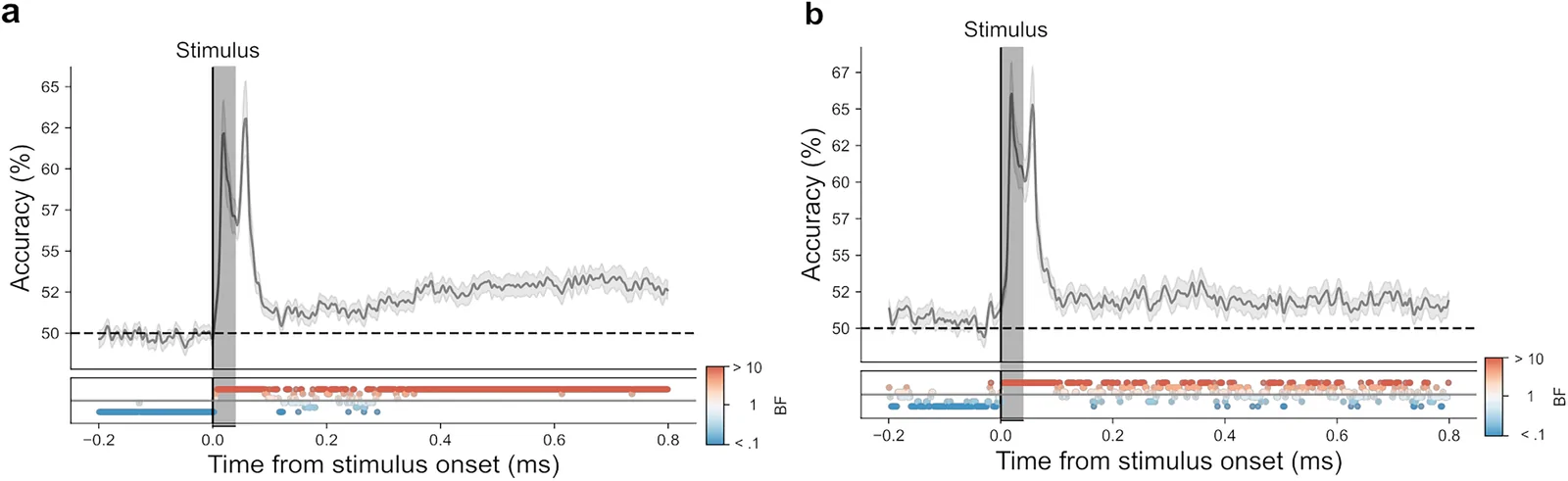
Above-chance decoding of finger location in passive and expected trials. Classifiers were trained and tested to distinguish patterns of neural activity evoked by stimulation of different fingers for (***a***) passive trials and (***b***) AS trials. The plotted results reflect the mean participant classification performance (*N* = 35) at corresponding training and testing timepoints; the shaded area represents the standard error. The gray vertical section shows the duration of the vibration stimulus (40 ms). The BFs below the plots indicate the timepoints at which there was substantial evidence in favor of the alternative hypothesis, i.e., decoding above-chance (dark red) and substantial evidence for the null hypothesis, i.e., chance level (dark blue).

We found that the classifier was able to accurately determine which finger was stimulated at a negligible latency after stimulus onset in both passive (Fig. 2*a*) and AS (Fig. 2*b*) trials. The initial decoding is likely too short for neuronal conduction delays, suggesting that very early above-chance decoding may be, at least partially, informed by an artifact of the mechano-vibratory stimulus. While early somatosensory ERPs have been reported (Dercksen et al., 2024), the EEG voltage patterns and scalp topography during these early decoding peaks indicate contamination by transient electrical or mechanical artifacts. Accordingly, we exclude the initial 100 ms following stimulation from our analysis window. Nevertheless, above-chance decoding persisted well beyond the stimulus duration. In the passive condition, there was sustained decoding of finger location lasting until 800 ms after stimulus onset. In the expected condition, there is strong evidence for finger location decoding up until ∼400 ms after stimulus onset. Following that point, decoding is slightly less consistent, although there is evidence of a finger location representation up until 800 ms. These results clearly demonstrate that multivariate classifiers were able to extract finger location information from the EEG signal.

### Stimulus omissions evoke a finger-specific PE

**Figure 3.**
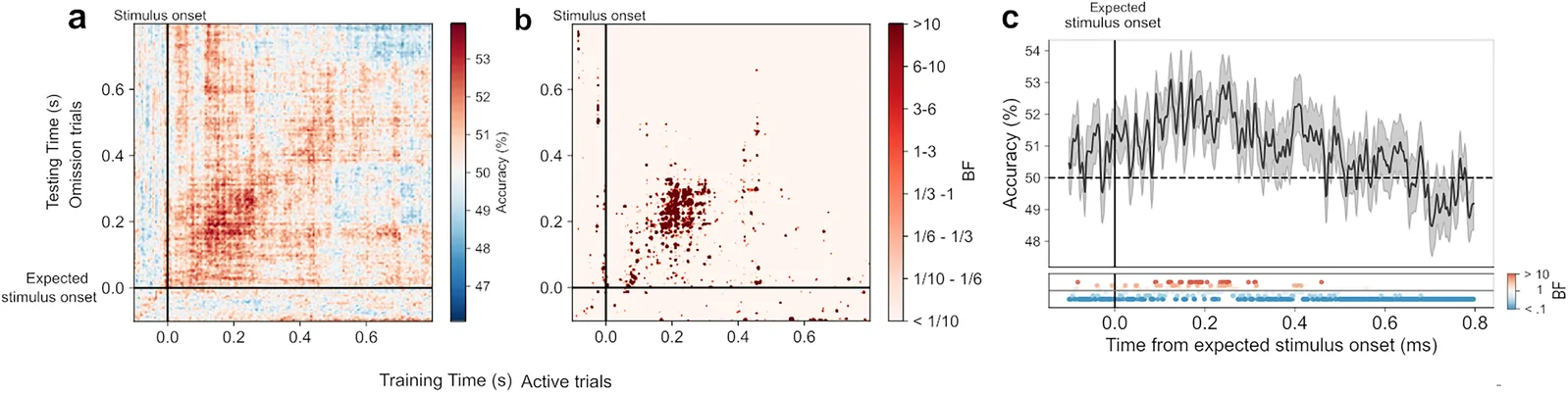
Finger-specific PE on AO trials (training on AS trials). Classifiers were trained on AS trials to distinguish patterns of neural activity evoked by stimulation of different fingers and subsequently tested on AO trials. ***a***, Temporal generalization matrix (TGM) showing above-chance decoding of finger location after omission onset. ***b***, The corresponding BFs. BFs show the strongest evidence (red) when training and testing on corresponding timepoints, i.e., along the diagonal. ***c***, Decoding along the diagonal of the TGM shown in ***a***. The group average (*N* = 35) decoding accuracy is plotted as a function of time from expected stimulus onset in omission trials; the shaded area represents the standard error. The BFs indicate the timepoints at which there was substantial evidence in favor of the alternative hypothesis, i.e., decoding above-chance (dark red) and substantial evidence for the null hypothesis, i.e., chance level (dark blue).

When training on AS trials and testing on AO trials, there was strong evidence that information about expected stimulation location was present in the neural activation patterns. This occurred between ∼120 and ∼250 ms following anticipated stimulus onset time, despite the absence of a physical stimulus (Fig. 3). This finding indicates that neural activation patterns evoked by the absence of expected finger stimulation are similar to stimulus-evoked activity. Importantly, this overlap occurred primarily along the diagonal of the temporal generalization matrix. This means that signals associated with the predicted stimulus unfolded at a similar time course as the actual processing of the stimulus.

**Figure 4.**
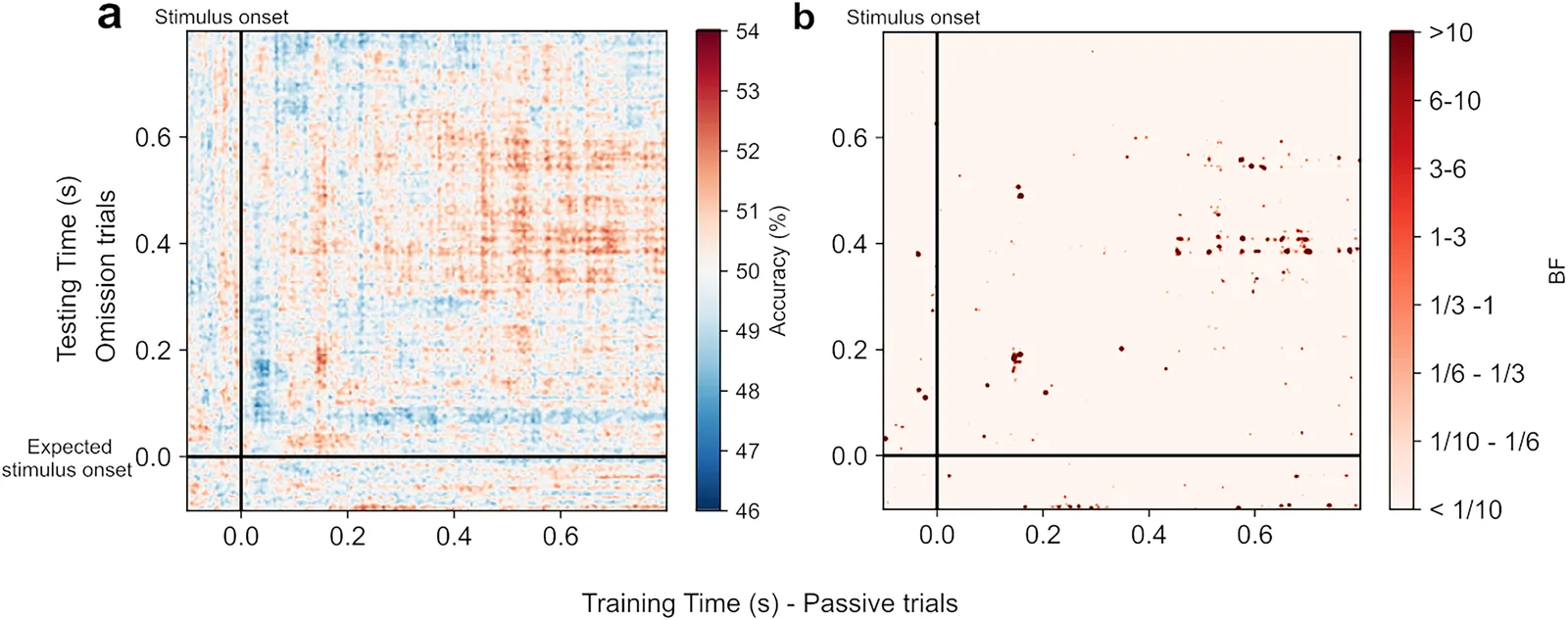
The omission response cannot be decoded when training on passive trials. Classifiers were trained on passive trials to distinguish patterns of neural activity evoked by stimulation to the index versus the ring finger and subsequently tested on AO trials. ***a***, Temporal generalization matrix (TGM) showing above-chance decoding (>50%) of finger location after omission onset. ***b***, The corresponding BFs. BFs show very little evidence for decoding of finger location, with mostly strong evidence for chance decoding (white).

In AE trials, participants had full control over stimulus onset time, making stimuli predictable in both time and location. Consequently, while the training data included stimulus-evoked activity, it likely also contained predictive signals since participants could anticipate the upcoming stimulus. This complicates the conclusions we can draw about the omission representation as it is not clear whether the trained classifier is relying on bottom–up signals generated by stimulus presentation or top–down activity arising from stimulus anticipation. To remove the influence of predictive signals and isolate the contribution of bottom–up stimulus activity, we trained our classifiers on passive trials in which the stimulus was unpredictable in both timing and location. Trained classifiers were tested on AO trials. We found almost exclusively at-chance decoding as shown in Figure 4. In other words, expected finger location could not be extracted from the omission response when training on data from passive trials. This may indicate that above-chance decoding shown in Figure 3 is driven by prediction-related activity.

### Exploratory analysis of omission response (training on passive)

**Figure 5.**
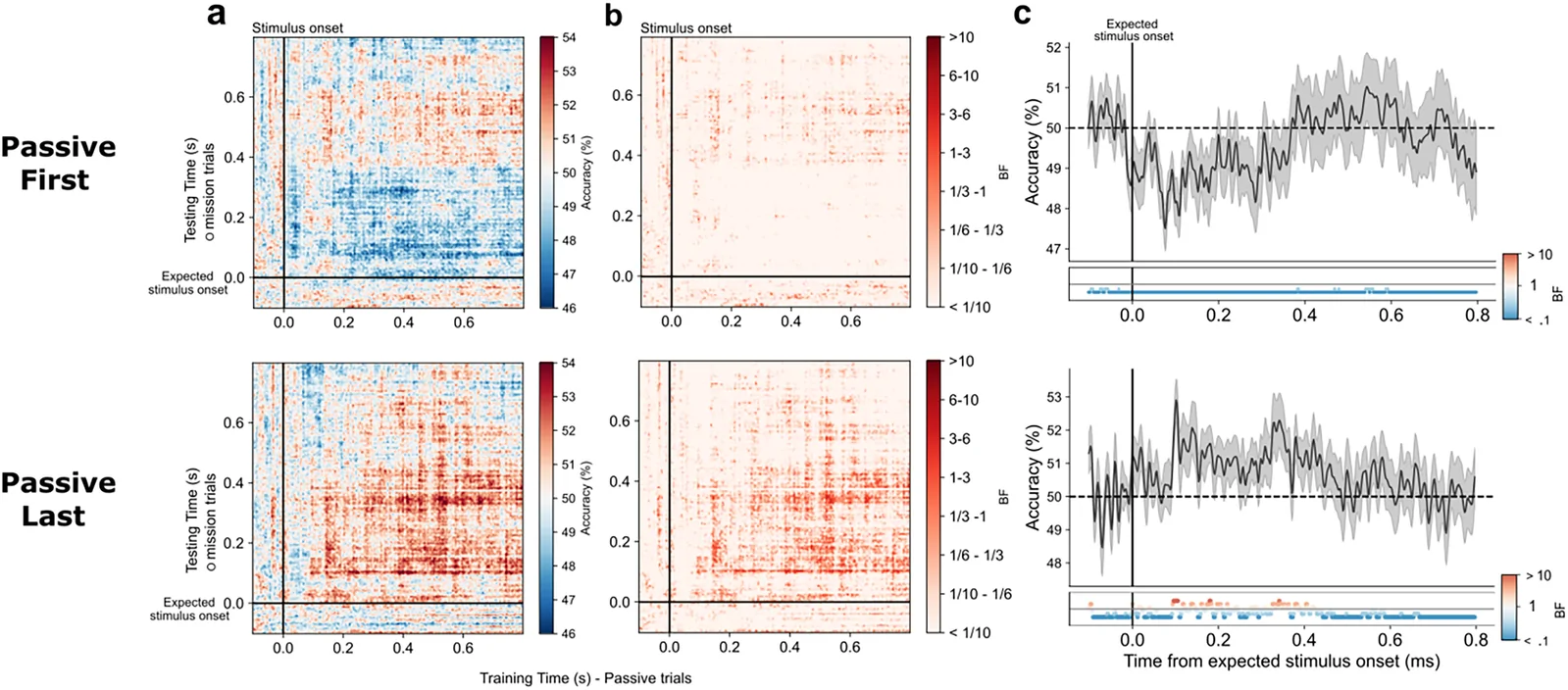
Block order affects decoding of omission response (training on passive trials). Classifiers were trained on passive trials to distinguish patterns of neural activity evoked by stimulation of different fingers and subsequently tested on AO trials. Results are grouped into participants who did the passive block before the active block (Passive First—top panel) and participants who did it after (Passive Last—bottom panel) ***a***, Temporal generalization matrices (TGMs) showing above-chance decoding of finger location after omission onset for Passive First (top panel) and Passive Last (bottom panel) groups. ***b***, The corresponding BFs. BFs show mostly strong evidence for chance decoding (white) in the Passive First group, while the Passive Last group show evidence for above-chance decoding (red) between 100 and 400 ms (using training timepoints between 200 and 800 ms). ***c***, The group mean (*N* = 35) decoding accuracy is plotted as a function of time from expected stimulus onset in omission trials averaged across training timepoints 200–800 ms after stimulus onset of passive trials. The shaded area represents the standard error. The BFs indicate the timepoints at which there was substantial evidence in favor of the alternative hypothesis, i.e., decoding above-chance (dark red) and substantial evidence for the null hypothesis, i.e., chance level (dark blue).

In an exploratory analysis, we tested for the effect of the block order when training on passive trials and testing on AO trials. Decoding results revealed above-chance decoding of finger location only for the “Passive Last” group. Strong evidence for above-chance decoding of finger location emerged ∼100 ms after omission onset at training timepoints between ∼200 and ∼800 ms (Fig. 5). This indicates that the omission response was similar to a late representation of the physical stimulus as presented on passive trials.

### Action induces predictive tactile stimulus templates

**Figure 6.**
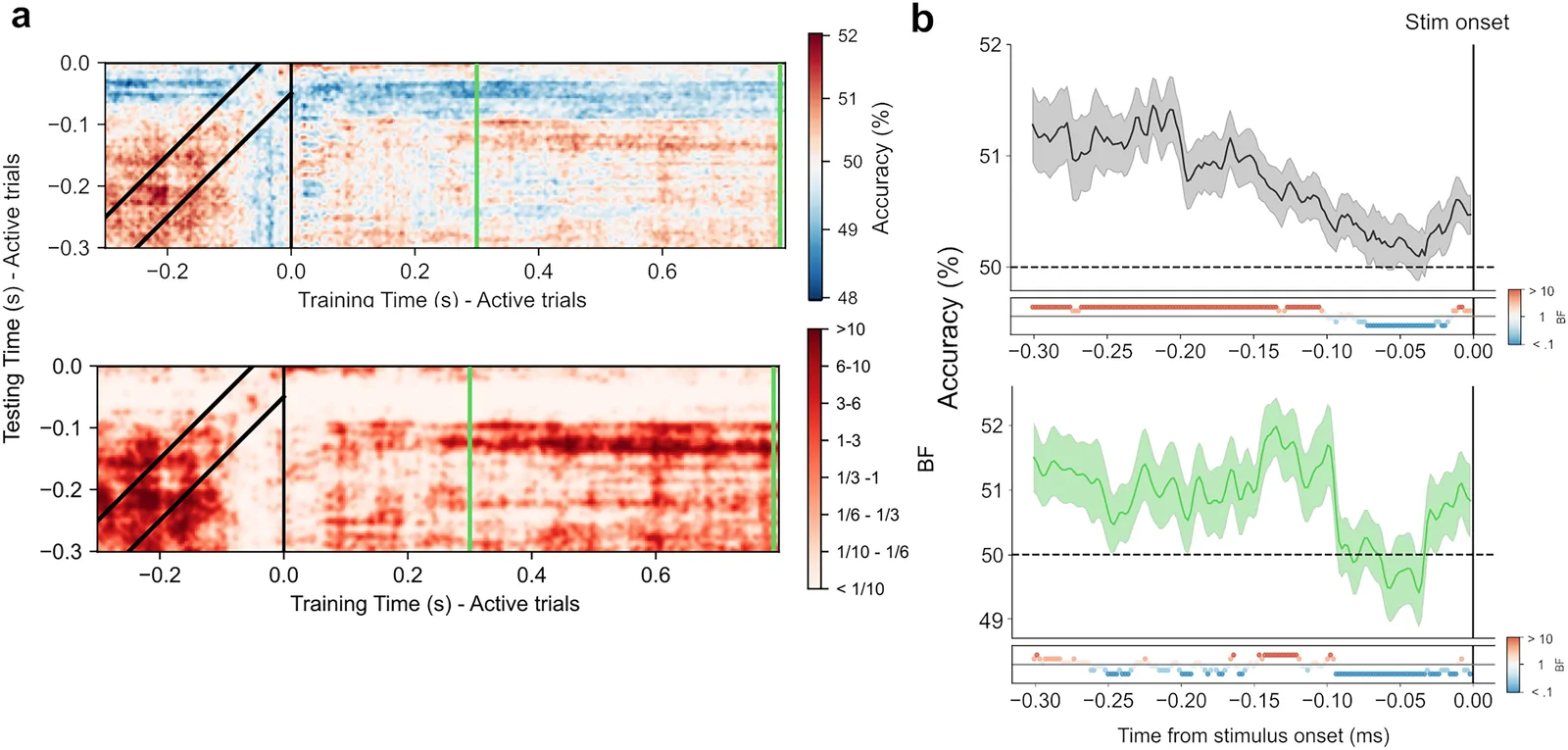
Finger-specific tactile template in the prestimulus period (training on expected trials). ***a*, Top panel**, Temporal generalization matrix (TGM) of classifiers trained on the time course of AS trials and tested on the prestimulus period of left-out AS trials. **Bottom panel**, The corresponding BFs for the TGM. BFs show the strongest evidence (red) when training and testing along the diagonal of the prestimulus period (illustrated by the black diagonal bands) and at training times from 300 to 800 ms after stimulus onset (illustrated by the vertical green bands). ***b*,** For each participant, we calculated the average classification performance in two bands (shown in ***a***). The black diagonal band reflects the similarity between corresponding training and testing times in the prestimulus period (**top panel**). The green band reflects the similarity between stimulus-evoked activity from 300 to 800 ms and prestimulus activity (**bottom panel**). The group mean (*N* = 35) decoding accuracy is plotted as a function of time before stimulus onset; the shaded area represents the standard error. The BFs below the plots indicate the timepoints at which there was substantial evidence in favor of the alternative hypothesis, i.e., decoding above-chance (dark red) and substantial evidence for the null hypothesis, i.e., chance level (dark blue).

Two interesting findings emerged when training on a subset of AS trials and testing on the prestimulus period of a separate subset of AS trials. Firstly, we found above-chance decoding along corresponding training and testing timepoints in the prestimulus period (Fig. 6*a*, data within black bands), and secondly, we found prestimulus decoding when using late poststimulus training data (Fig. 6*a*, data within green bands). For each participant, we calculated the average classification score within the bands. For the diagonal band in the prestimulus period, there was strong evidence for above-chance decoding of finger location from 300 to 100 ms before stimulus onset (Fig. 6*b*, top panel). This means that there was information about the expected stimulation location in prestimulus EEG activity. In addition, we calculated classification performance in the prestimulus period averaging over training times from 300 to 800 ms poststimulus onset. The strongest evidence for above-chance classification performance was between ∼150 and 130 ms before stimulus onset (Fig. 6*b*, bottom panel). This suggests that starting at ∼150 ms the predictive representation of finger location becomes similar to the late representation of a physically presented tactile stimulus.

## Discussion

To investigate action-effect tactile predictions, participants made index finger movements paired with stimulation to the index or ring finger of the opposite hand. On rare trials, tactile stimulation was omitted. Thus, within each block, participants formed a location-specific prediction, with stimulus timing determined by their movement. Using EEG and multivariate decoding, we found that in the absence of bottom–up input, we could decode expected finger location, effectively capturing a location-specific prediction–error response. In addition, we found that prestimulus activity contained information about the expected stimulation site, evidence of an anticipatory tactile template.

### The omission response

Given that omission-related responses cannot be explained by feedforward activity, any information about stimulus identity likely reflects a purely prediction-related signal (Wacongne et al., 2012). We found finger-specific decoding ∼120–250 ms after omission, consistent with auditory ERP omission responses (Todorovic et al., 2011; Wacongne et al., 2011; Sanmiguel et al., 2013a,b). The omission response can be explained by predictive coding whereby the repeated coupling of movement and finger-specific stimulation enables formation of a sensory prediction, activating neurons tuned toward the expected stimulation location (Reznik et al., 2015; Schneider and Mooney, 2018; Pazen et al., 2020).

While above-chance omission decoding supports the presence of stimulus-specific predictions, distinguishing whether this reflects the prediction or PE remains challenging. Predictive coding models assume that, on omission trials, the prediction (e.g., index finger vibration) is subtracted from the bottom–up input (no vibration), producing a negative PE. Negative PEs arise when the actual input is weaker than predicted and may be neurally implemented through bottom–up inhibition of the relevant neurons (Keller and Mrsic-Flogel, 2018). This inhibition likely suppresses activity of neurons encoding the expected stimulus which may manifest as at- or below-chance decoding because the classifier is effectively decoding the inverse of its training pattern. Instead, we observed above-chance decoding suggesting that omission decoding may reflect the sensory prediction of the absent stimulus or perhaps a combination of prediction-related and error-related activity (Wacongne et al., 2012). Thus, while we can conclude that tactile representations are predictively evoked by movement, further research is needed to identify the precise neural origins of the omission response.

The fact that omission activity could be decoded by classifiers trained on physically presented stimuli indicates that the neural prediction resembles actual tactile input. Across two analyses, we trained classifiers on active-stimulus (AS) trials and on passive trials (“Passive Last”), testing both on active-omission (AO) trials with somewhat different results. An interesting difference is the training times at which above-chance decoding emerged. Training on AS trials produced diagonal decoding, indicating similar patterns at corresponding timepoints. When training on passive trials, late tactile patterns (∼400 ms poststimulus) were most similar to omission activity. This difference is likely driven by predictability. In passive trials, the tactile stimulus was unpredictable in terms of both finger location and onset time while AS trials had fully predictable action-effect contingencies. Prediction formation in both training (AS) and test sets (AO) may explain the similarity of representations at corresponding timepoints.

When training on passive trials, omission decoding occurred only in the “Passive Last” group. This aligns with ideomotor theory, which posits that repeated exposure to actions and their sensory effects establishes a bidirectional relationship where both the action and the consequence are represented by a common code (Eisner and Hommel, 2001; Shin et al., 2010; Hommel, 2013). Because participants in the “Passive Last” group had already encoded the action-effect relationship, the passive tactile stimulus subsequently activated neural patterns that overlapped with the action-related representation of the same effect. In the “Passive First” group, decoding trended toward below-chance at omission, which may reflect an inhibitory, prediction error-like response to the unexpected absence of touch before any learned action-effect associations had formed. This is an interesting finding as the movement was equally paired with stimulation to the index and ring finger. This suggests that multiple action-effect pairings can be held simultaneously and flexibly employed, perhaps adapting as the context changes.

### The predictive stimulus template

Our results support the idea that predictions preactivate stimulus-specific neural ensembles (Ekman et al., 2017; Kok et al., 2017; Ody et al., 2023; 2024). While prior work has shown action-dependent modulation of the sensory cortex preceding expected outcomes (Reznik et al., 2021), our results show that these predictive signals contain stimulus-specific information about upcoming events. Training and testing on the prestimulus period (AS trials) revealed a sustained representation of upcoming finger location from 300 to 100 ms before stimulus onset. Crucially, this decoding cannot be attributed to the movement, which was identical regardless of stimulation location. From ∼150 to 100 ms before stimulus onset, the predictive representation became more stimulus-like, evidenced by successful decoding using late (300–800 ms) poststimulus training patterns. Decoding with such late timepoints likely indexes higher-order somatosensory representations consistent with predictive and integrative processes (Smit et al., 2023), rather than low-level SI activity. Recent fMRI work, using a similar design, found higher decoding for congruent versus incongruent action-effects in the occipital and occipitotemporal cortex, indicating a sharpened representation driven by expectation (Yon et al., 2018). The present results complement this by revealing the temporal dynamics of such predictions, showing that finger-specific tactile representations emerge prior to movement.

In our analysis, we epoched around stimulus onset for temporal precision but anticipatory activity likely emerges long before movement onset. This interpretation aligns with recent work showing that the RP, beginning 1–2 s before movement, encodes identity-specific predictions (Gärtner et al., 2025). Movement onset in our study was defined by a 10 mm downward displacement of the finger. On average, participants required ∼30 ms to move the remaining 5 mm before stimulation. Even assuming constant velocity, movement initiation would precede stimulation by ∼90 ms, an overestimate given natural acceleration–deceleration profiles (Morasso, 1981; Abend et al., 1982; Uno et al., 1989). Thus, decoding 100 ms prior to stimulus onset likely reflects premovement predictive activity.

The decoding drop in the final 100 ms before stimulus onset should be interpreted cautiously, as baseline correction may have attenuated identity-specific activity during this interval. In addition, given that stimulation location was constant within blocks, the present design cannot fully dissociate anticipatory prediction from finger-specific attention, and both processes may contribute to the observed prestimulus decoding.

### Nonmotor prediction mechanisms

Action-effect predictions are often described within a FM framework (Wolpert and Miall, 1996). While self-generated touch is no exception (Blakemore et al., 1998), our findings violate some tenets associated with an FM. First, internal predictions are thought to update gradually (Rummell et al., 2016; Schneider et al., 2018), yet our task required rapid and flexible switching of action-effect mappings across blocks. Despite this, we found reliable decoding of finger location, demonstrating a quick reconfiguration of predictions. This differs from animal studies where attenuation emerges only after extended training (Rummell et al., 2016; Schneider et al., 2018), suggesting that motor-based FMs may not fully explain rapid predictive updating.

Another important attribute of the FM is that predictions depend on the likelihood that a sensation is self-generated. Previous behavioral studies have shown that attenuation of self-touch depends on whether self-touch is physically plausible (Bays and Wolpert, 2008; Kilteni and Ehrsson, 2017). When hand positioning makes self-touch impossible, the sensory attenuation effect is significantly reduced (Bays and Wolpert, 2008) or absent entirely (Kilteni and Ehrsson, 2017). In our setup, while the moving and passive hands were relatively close to one another, the distance did not allow for real self-touch. Nevertheless, we found strong evidence for prediction formation of stimulation location. This suggests that the FM may be more flexible than originally conceived or that our findings can be explained by nonmotor mechanisms.

Sensory predictions also arise from learned statistical regularities, producing attenuation effects in both visual (Summerfield and Koechlin, 2008; Alink et al., 2010; Kok et al., 2012; Richter et al., 2018) and auditory (Lange, 2009; Hughes et al., 2013; Schröger et al., 2015) modalities. Some have suggested that the motor system may be part of a more general prediction system that guides both perception and action (Thomas et al., 2022; Press et al., 2023). This is in line with theoretical accounts such as active inference (Friston et al., 2009, 2011; Friston, 2011; Adams et al., 2013) and ideomotor theory (Elsner et al., 2001; Shin et al., 2010; Hommel, 2013). While these posit that predictions accompany action, motor-specific FMs are not the driving force. It is possible that our results reflect such domain-general predictive processes. Determining the relationship between motor and nonmotor predictions remains challenging because, as expected, nonmotor paradigms typically do not involve action. Our study helps bridge these domains by manipulating predictions within the context of self-generated action (Thomas et al., 2022). Future work should investigate how nonmotor predictions influence motor–perception interactions and establish whether the same neural mechanisms generate predictions. Future studies could adapt this design to include a passive-omission condition with predictable stimulation timing, allowing direct comparison of omission-related responses arising under motor versus purely sensory predictive contexts. This comparison would help clarify whether predictive templates are specific to action-effect learning or reflect a more general, modality-independent mechanism.

## Data Availability

All experiment scripts, preprocessed data, and analysis code will be made available on the Open Science Framework (https://osf.io/yw8sa/).
